# Is it necessary to perform a diagnostic hysteroscopy before the first embryo transfer?—A retrospective study

**DOI:** 10.3389/fmed.2025.1690944

**Published:** 2025-11-12

**Authors:** Jian-ye Fang, Yang-ying Xu, Hui-min Zhang, Duan Li, Ze-wei Yu, Cui-fang Hao

**Affiliations:** 1 Center for Reproductive Medicine, Women and Children's Hospital, Qingdao University, Qingdao, China; 2 Branch of Shandong Provincial Clinical Research Center for Reproductive Health, Qingdao, China; 3 College of Medicine, Qingdao University, Qingdao, China

**Keywords:** hysteroscopy, IVF, embryo transfer, infertility, clinical pregnancy rate

## Abstract

**Objective:**

This study aimed to assess the impact of performing diagnostic hysteroscopy prior to the first *in vitro* fertilization (IVF) cycle on clinical pregnancy rates and live birth rates.

**Methods:**

A retrospective descriptive study was conducted from October 2019 to March 2023 at Qingdao Women and Children's Hospital, China. The study population included women under 45 years old with ultrasonographically normal uterine cavities who were undergoing their first fresh embryo transfer through *in vitro* fertilization (IVF) or intracytoplasmic sperm injection (ICSI). Primary outcomes included: (1) prevalence of abnormal uterine findings detected by hysteroscopy, and (2) comparative analysis of reproductive outcomes between hysteroscopy and non-hysteroscopy groups.

**Results:**

Among patients undergoing hysteroscopy, 49.63% patients exhibited abnormal uterine findings, with endometrial polyps being the most common pathology (30.03%). A significantly lower rate of good-quality embryos was observed in the hysteroscopy group compared to the non-hysteroscopy group (50.38% vs. 75.11%, *p* < 0.05). After adjusting for embryo quality, age, BMI, AMH, duration of infertility, and endometrial thickness, multivariable analysis confirmed that the hysteroscopy group had a significantly higher clinical pregnancy rate (OR: 1.51, 95% CI: 1.142–1.997, *P* = 0.004) compared to the non-hysteroscopy group. In the subgroup, the clinical pregnancy rate in these two groups (the endometrial polyp group 63.49%, *p* = 0.014; the chronic endometritis group 64.12%, *p* = 0.032) was significantly higher. No statistically significant difference in live birth rate was observed between the groups.

**Conclusions:**

Diagnostic hysteroscopy effectively identifies and facilitates treatment of intrauterine abnormalities in IVF/ICSI candidates to optimize endometrial receptivity. And performance of hysteroscopy prior to IVF is significantly associated with increased clinical pregnancy rates. These findings support the recommendation for pre-transfer hysteroscopic evaluation in the first embryo transfer cycles.

## Introduction

The success of embryo implantation is a complex condition that depends on numerous mechanisms regarding its occurrence and evolution. Implantation failure could be due to a variety of factors, including embryo quality, uterine receptivity, and other unexplained cases (1). Notably, endometrial receptivity is estimated to account for up to two-thirds of the embryo implantation failure, with uterine cavity abnormalities—such as endometrial polyps, chronic endometritis, or intrauterine adhesions—serving as a primary compromising factor (2, 3). Therefore, evaluating and assessing the uterine cavity constitute some of the most fundamental and crucial steps in the examination of infertile women. This is because the normal anatomy of the uterine cavity is indispensable for the endometrium's physiological function, which in turn is vital for the occurrence of pregnancy. Clinically, we commonly use ultrasound to detect uterine cavity lesions. Sometimes, in order to further find the uterine cavity lesions, three-dimensional ultrasound can also be performed. Some researchers point that three-dimensional ultrasound with advantages of giving better spatial orientation, non-invasive, pain free, can serve as an alternative to hysteroscopy for screening endouterine lesions (4, 5). Moreover, this research reveals that there is no statistically significant edge of hysteroscopy compared to three-dimensional ultrasound, for 50% of patients with a normal uterine cavity, hysteroscopy may be regarded as unnecessary (5). However, three-dimensional ultrasound, saline infusion sonohysterography, and hysterosalpingography might not be adequate to detect and treat minor lesions within the uterine cavity, such as endometrial congestion, tiny endometrial polyps, and mild intrauterine adhesions (6, 7). We need hysteroscopy to observe the endometrium more directly and accurately. In addition, hysteroscopy is advantageous as it can be used to execute endometrial biopsy and treatment. However, a recent study (8) suggest that performing hysteroscopy before assisted reproductive technology (ART) procedures does not improve the pregnancy outcomes. Therefore, the efficacy of hysteroscopy prior to assisted reproductive technique (ART) remains controversial.

In our study, pregnancy outcomes of the first IVF cycles were retrospectively analyzed when the embryo transfer was performed that applied with or without hysteroscopy to evaluate the pre-IVF uterine cavity.

## Material and methods

### Patients

This study was conducted on a retrospective analysis of patients who performed fresh embryo transfer through *in vitro* fertilization (IVF) or intracytoplasmic sperm injection (ICSI) for the first time due to infertility from Qingdao women and children's Hospital, Shandong, China.

We collected a total of 2,014 patients under 45 years-old with infertility who had normal uterine cavity examined by ultrasound from October 2019 to March 2023. The inclusion criteria were formulated using infertility as the primary diagnosis and hysteroscopy as the diagnostic or operative procedure.

Exclusion criteria: (1) Age >45 years-old, or age < 20 years-old. (2) Patients with diagnosed uterine anomalies. (3) Cases of premature ovarian failure. (4) History of any previous uterine surgery and IVF/ICSI treatment. (5) Cervical stenosis and inability to perform hysteroscopy.

### Hysteroscopy and treatment/management

Hysteroscopy was performed during the early proliferative phase in the outpatient clinic with anesthesia using by a 3.5 mm Olympus hysteroscope with a 30° view. Saline distension medium was used. Patients were operated only for 15–30 min and no complications were experienced. The hysteroscopic images of all patients were jointly diagnosed by two experienced doctors. Endometrial biopsy was performed in all patients for pathological verification.

Endometrial polyps or intrauterine adhesions detected during surgery were excised with micro-scissors or micro-forceps. In cases requiring adhesiolysis, postoperative estrogen therapy was administered to promote endometrial regeneration.

Hysteroscopic evaluation revealed characteristic features of chronic endometritis, including endometrial strawberry appearance, focal or diffuse endometrial hyperemia, micro-polyps, and stromal edema (9, 10). The histopathological diagnosis of CE was confirmed by the presence of ≥1 plasma cell or CD138-immunopositive cells per 10 high-power fields (HPFs) (11). During the procedure, CE-associated lesions such as micro-polyps and hyperemic endometrium were precisely resected using micro-scissors. Postoperatively, patients routinely received a 14-day course of doxycycline as anti-inflammatory therapy (12).

### The ovarian stimulation protocol and embryo transfer

The ovarian stimulation protocol was individualized based on the patient's age and ovarian reserve, typically employing either an antagonist protocol or a Long GnRH agonist protocol. Gonadotropins were administered at individualized doses based on patient response, with human chorionic gonadotropin (hCG, 6,000–8,000 IU) triggering final oocyte maturation.

In long protocols, the GnRH agonist is administered either on the day 2 of menstruation, or during the luteal phase. A GnRH antagonist was added when dominant follicle reached ≥12–14 mm, or LH >10 IU/L or LH doubled baseline, or E2 ≥1,100 pmol/L, and continued daily until hCG administration. Following oocyte retrieval and fertilization, embryos were transferred on day 3 or day 5.

### Criteria for embryo transfer cancellation

Criteria for embryo transfer cancellation: (1) On the day of HCG administration, E2 > 15,000 pmol/L, and the number of follicles ≥14 mm in diameter in both ovaries is ≥15. (2) The number of follicles ≥12 mm in diameter in both ovaries is ≥20. (3) Any of the following ultrasound findings: endometrial thickness ≤ 0.6 cm or thickness >1.6 cm with heterogeneous or mass-like echogenicity, without ruling out endometrial lesions such as intrauterine adhesions or endometrial polyps; untreated communicating hydrosalpinx; endometrial separation or intrauterine fluid accumulation, or persistent vaginal bleeding for more than 8 days with endometrial recovery time < 7 days. (4) Serum progesterone level ≥5 nmol/mL on the day of HCG administration. (5) Patients with asynchronous endometrial development after ≤ 6 days of Gn medication. (6) Patients undergoing PGT (preimplantation genetic testing). (7) Patients with poor ovarian reserve, or those with uterine fibroids or adenomyosis planning for surgery, who are undergoing embryo accumulation. (8) Patients undergoing fertility preservation. (9) Incomplete preoperative test results or abnormal conditions requiring treatment, such as vaginitis, acute genitourinary infection, or acute infectious diseases.

### Criteria for a good embryo

Criteria for a good embryo day 3 include: (1) Origin from a normally fertilized zygote. (2) 7 to 9 cells on day 3. (3) Cell size consistent with the developmental stage. (4) Less than 10% fragmentation. (5) No multinucleation (13).

Criteria for a high-quality blastocyst: must be Gardner Stage 3 or above, excluding any grade C for inner cell mass or trophectoderm (14).

### Groups

**Figure 1:**
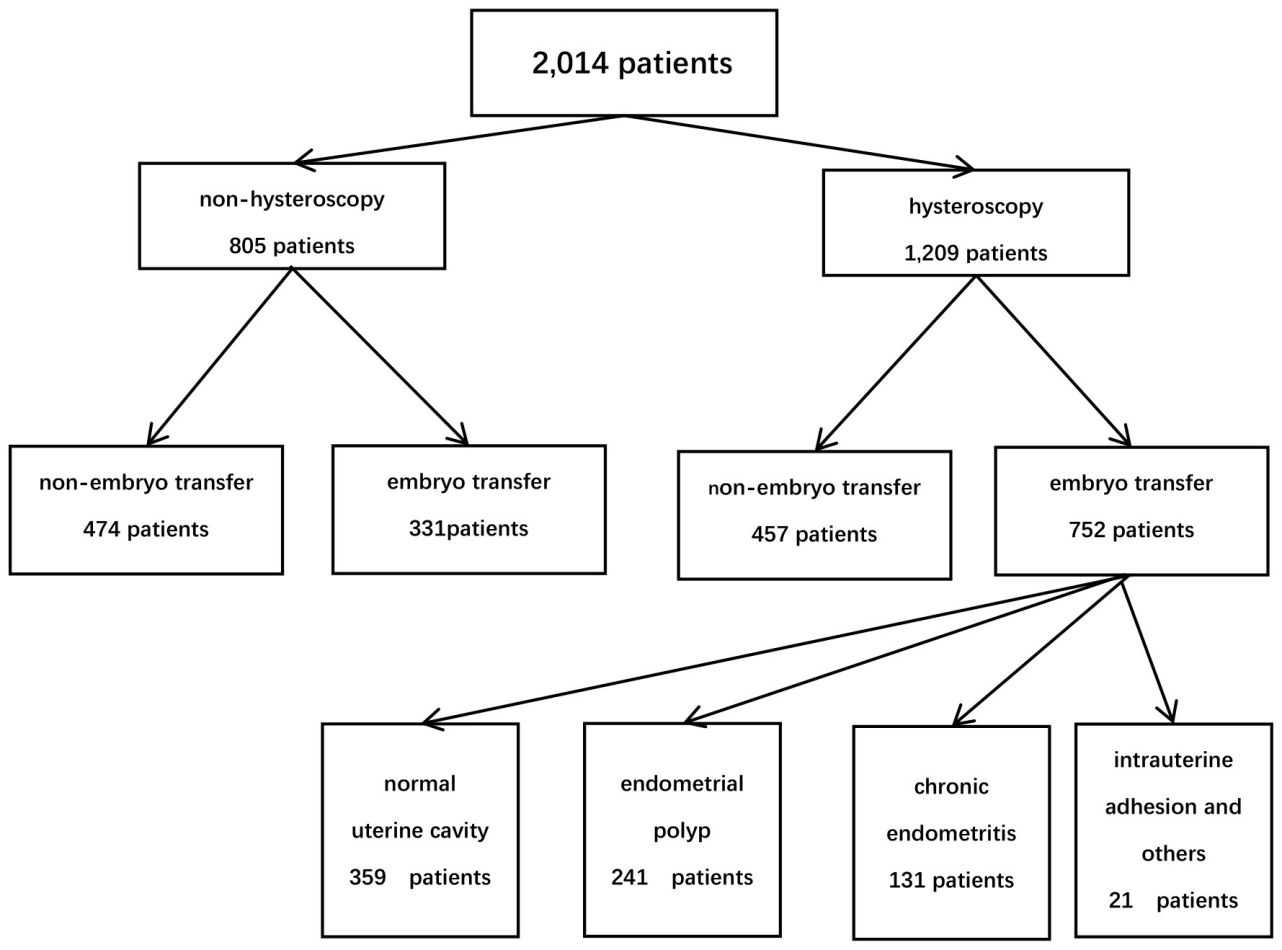
Flow chart of all participants' enrollment and group allocation.

Based on the patients' financial situation and personal preferences, they were divided into two groups: the hysteroscopy group and the non-hysteroscopy group. Among them, 1,209 patients performed hysteroscopy, while 805 patients did not. In the hysteroscopy group (study group), 752 patients proceeded with embryo transfer, whereas 331 patients in the non-hysteroscopy group underwent embryo transfer (control group). According to the hysteroscopic findings and endometrial pathology with immunohistochemistry, patients were divided into four groups: normal uterine cavity (*n* = 359), endometrial polyp (*n* = 241), chronic endometritis (CE) (*n* = 131), intrauterine adhesion and others (*n* = 21) (Figure 1).

### Outcomes

The proportion of normal and abnormal diagnosis in hysteroscopy was analyzed. The pregnancy outcomes of fresh cycle embryo transplantation were compared between patients who underwent hysteroscopy and those who did not. The baseline (age, BMI, AMH, infertility years), endometrial thickness, good embryo rate, clinical pregnancy rate, live birth rate and abortion rate were compared among the groups. A further multivariable binary logistic regression analysis was conducted to assess the factors associated with pregnancy outcomes.

### Statistical analysis

All parameters were evaluated and processed in IBM SPSS Statistics software, version 29. The values of the continuous variables are expressed herein as mean (SD) and absolute (%) frequencies. The between-group differences were compared by using the independent samples *t*-test and chis-quare test. Statistical significance was defined as *P* < 0.05. A multivariable binary logistic regression analysis was performed to identify the independent factors associated with pregnancy outcomes. Statistical results were generated using pie charts and tables, adding to the processing graphs obtained with Microsoft Office Excel 2007.

## Results

**Figure 2:**
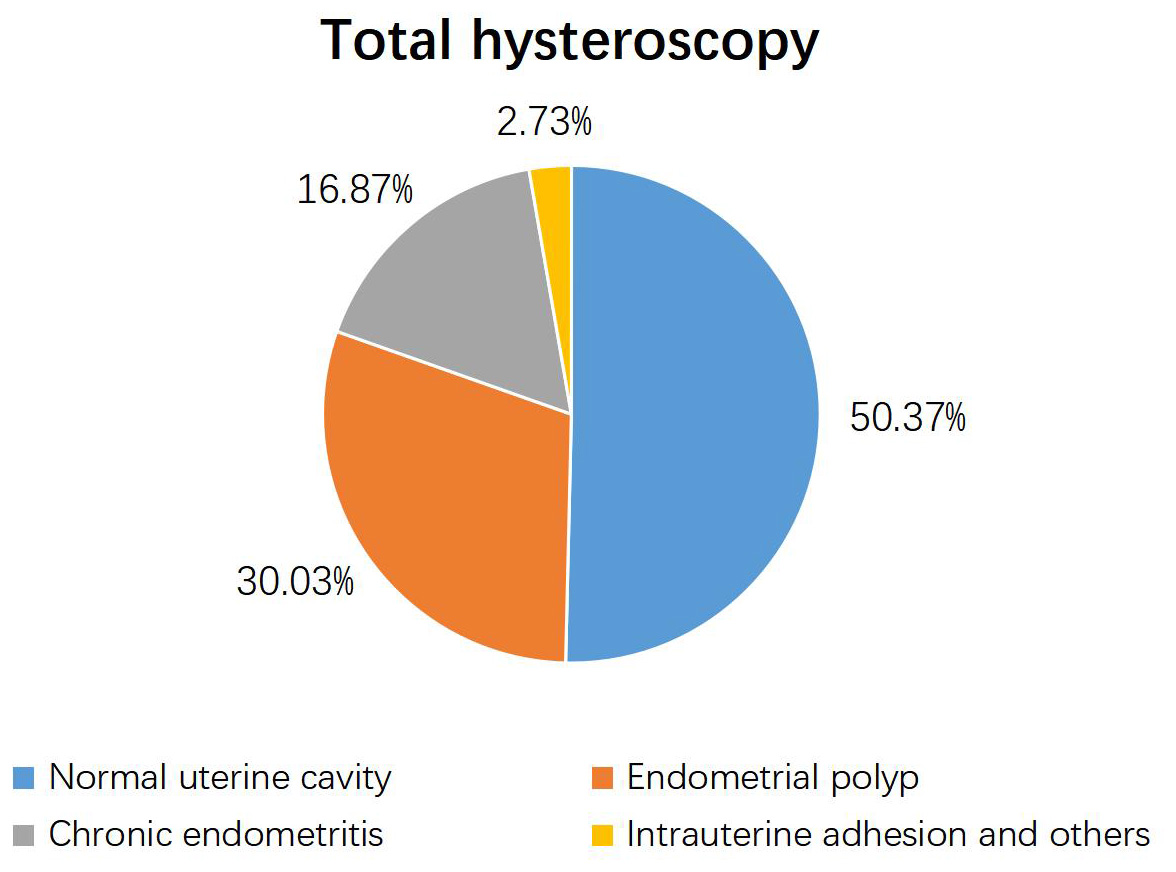
Proportion of normal and abnormal hysteroscopy.

Among the 1,209 study participants, hysteroscopy identified uterine cavity pathologies in 605 women (49.63%) and a normal uterine cavity in 600 women (50.37%). Endometrial polyps were the most prevalent pathological finding, detected in 30.03% of case (Figure 2).

Among 1,083 patients who underwent embryo transfer, 752 (69.4%) received hysteroscopy before transplantation, while 331 (30.6%) did not. There were no differences in age, BMI, AMH, or infertility years between the hysteroscopy group and the non-hysteroscopy group. In the subgroup analysis, due to the limited sample size (*n* = 21) in the intrauterine adhesion (IUA) subgroup, patients with IUA in the study group demonstrated significantly lower AMH levels compared to the control group (0.91 ± 0.47 ng/mL vs. 3.53 ± 2.80 ng/mL, *p* = 0.001; Table 1).

**Table 1:** Characteristics of embryo transfer patients.

**Group**	**No. of patients**	**Age (years)**	**BMI (kg/m^2^)**	**AMH (ng/ml)**	**Infertility years**
Non-hysteroscopy	331	32.70 ± 4.38	23.14 ± 3.46	3.53 ± 2.80	3.49 ± 2.51
Hysteroscopy	752	33.06 ± 4.31	23.14 ± 3.21	3.59 ± 2.72	3.40 ± 2.33
Normal uterine cavity	359	33.36 ± 4.50	23.21 ± 3.10	3.51 ± 2.76	3.40 ± 2.32
Endometrial polyp	241	32.73 ± 4.18	23.06 ± 3.31	3.61 ± 2.56	3.58 ± 2.35
Chronic endometritis	131	32.63 ± 3.92	22.93 ± 3.32	4.07 ± 2.85	3.18 ± 2.34
Intrauterine adhesion and others	21	34.48 ± 4.21	24.21 ± 3.29	0.91 ± 0.47^*^	2.71 ± 2.10

^*^p < 0.05, compare with control.

The clinical pregnancy rate in the hysteroscopic group was slightly higher than that in the non-hysteroscopic group. While no statistically significant differences were observed in clinical pregnancy rates (58.24% vs. 53.17%, *p* = 0.12) or live birth rates (47.34% vs. 44.11%, *p* = 0.326) between the hysteroscopy and non-hysteroscopy groups, the hysteroscopy group demonstrated a significantly lower good-quality embryo rate (50.38% vs. 75.11%, *p* < 0.05). In the hysteroscopic subgroup, the clinical pregnancy rate of the chronic endometritis was the highest, followed by the endometrial polyp group, and the intrauterine adhesion group was the lowest. Compared to the non-hysteroscopy group, although the good-quality embryo rate was lower in both the endometrial polyp and the chronic endometritis group, the clinical pregnancy rate in these two groups (the endometrial polyp group 63.49%, *p* = 0.014; the chronic endometritis group 64.12%, *p* = 0.032) was significantly higher than that in the non-hysteroscopy group. The rate of good-quality embryos in the endometrial polyp group and the endometritis group was significantly lower than that in the non-hysteroscopy group the endometrial polyp group 49.08%, *p* < 0.001; the chronic endometritis group 43.96%, *p* < 0.001. The miscarriage rate of these two groups was also slightly higher than that in the non-hysteroscopy group, but the difference was not statistically significant. No statistically significant difference in the live birth rate was observed among all groups. No statistically significant difference in endometrial thickness was observed between the hysteroscopy and non-hysteroscopy groups. In subgroup analyses, both the normal uterine cavity and intrauterine adhesion (IUA) subgroups showed significantly thinner endometrium compared to the non-hysteroscopy group (*p* < 0.05), but all measurements remained within the normal range (7–12 mm) (Table 2).

**Table 2:** Comparison of outcomes between embryo transfer groups.

**Group**	**No. patients of embryo transferred**	**Endometrial thickness (mm)**	**Good embryo rate D3**	**Good blastocyst rate**	**No. patients of clinical pregnancy**	**Clinical pregnancy rate**	**No. patients of miscarriage**	**Miscarriage rate**	**No. patients of live birth**	**Live birth rate**	**No. patients of ectopic pregnancy**
Non-hysteroscopy	331	10.38± 1.72	75.11%	92.94%	176	53.17%	25	14.20%	146	44.11%	5
Hysteroscopy	752	10.16± 1.56	50.38%^*^	90.57%	438	58.24%	75	17.12%	356	47.34%	8^#^
Normal uterine cavity	359	9.95 ± 1.46^*^	53.50%^*^	86.96%	190	52.92%	25	13.16%	160	44.57%	6^#^
Endometrial polyp	241	10.57± 1.60	49.08%^*^	90.91%	153	63.49%^*^	30	19.61%	122	50.62%	1
Chronic endometritis	131	10.19± 1.42	43.96%^*^	95.24%	84	64.12%^*^	17	20.24%	66	50.38%	1
Intrauterine adhesions and others	21	8.70 ± 2.25^*^	52.00%^*^	100%	11	52.38%	3	27.27%	8	38.10%	0

^*^p < 0.05, compare with control; ^#^Heterotopic pregnancy: one case.

Hysteroscopy before IVF significantly increases the clinical pregnancy rate (OR: 1.51, 95% CI: 1.142–1.997 *P* = 0.004), compared with the control group. Age is significantly negatively correlated with pregnancy outcomes (OR: 0.93, 95% CI: 0.904–0.96, *P* < 0.001), whereas high-quality embryos are significantly positively correlated with pregnancy outcomes (OR: 1.881, 95% CI: 1.407–2.515, *P* < 0.001). No significant differences in the BMI, AMH, infertility years and endometrial thickness were observed between groups (Table 3).

**Table 3:** Association among hysteroscopy, age, BMI, AMH, infertility years, good embryo, and clinical pregnancy (multivariable binary logistic regression analysis).

**Reference group: non-hysteroscopy**				
**Variable**	**B**	**OR**	**95%CI for OR**	**P-value**
Hysteroscopy	0.412	1.51	1.142; 1.997	0.004
Age	−0.071	0.932	0.904; 0.96	< 0.001
BMI	0.032	1.032	0.993; 1.073	0.107
AMH	−0.041	0.96	0.916; 1.006	0.088
Infertility years	−0.028	0.972	0.922; 1.025	0.291
Endometrial thickness	0.014	1.014	0.953; 1.08	0.651
Good embryos	0.632	1.881	1.407; 2.515	< 0.001

## Discussion

Hysteroscopy is widely acknowledged as the “gold standard” test for assessing the uterine cavity (15). While first-line imaging like transvaginal ultrasound is recommended for detecting endometrial polyps (Level B) (16), it may miss small lesions. Although three-dimensional ultrasound offers advantages over conventional methods, its diagnostic accuracy (sensitivity: 68.2%; specificity: 91.5%) remains inferior to hysteroscopy (7), which demonstrates the highest diagnostic precision in infertile patients with suspected polyps (Level B) (16). Hysteroscopy not only works as a direct and accurate diagnostic tool, but also provides a therapeutic approach for identified pathologies, including intrauterine abnormalities, submucous myomas, and adhesions—all of which are potential major contributors to implantation failure. Notably, our study revealed that while a half of the patients showed no abnormal lesions by ultrasound, hysteroscopy still detected subtle uterine cavity lesions. Our result agrees with prior research that used hysteroscopy as the gold standard after 3D ultrasound and identified a normal uterine cavity in 50% of the patients (5). The most prevalent findings were endometrial polyps and endometritis, both of which are known to compromise embryo implantation success and reducing pregnancy rates (17–19). Hysteroscopy not only enables direct visualization and diagnosis of such intrauterine pathologies but also permits immediate intervention, such as polypectomy, to eliminate factors detrimental to implantation. Supporting this approach, a recent study confirmed that hysteroscopic polypectomy significantly improves IVF outcomes—in terms of both clinical and total pregnancy rates—in women with suspected endometrial polyps and a history of prior IVF failure (20). Furthermore, a recent review underscores the importance of diagnostic hysteroscopy and endometrial receptivity assessment as key strategies to improve implantation rates (21).

Our study found that there was no significant difference in pregnancy rates between the hysteroscopy and non-hysteroscopy groups. However, the non-hysteroscopy group exhibited a significantly higher good-quality embryo rate. Notably, if embryo quality had been comparable between the two groups, the hysteroscopy group might have a higher pregnancy rate than the control group. Therefore, a multivariable regression analysis was performed in our study, and the results showed that a 1.51-fold higher odds of clinical pregnancy in the hysteroscopy group compared to the non-hysteroscopy group (OR: 1.51, 95% CI: 1.142–1.997, *P* = 0.004). This observation aligns with existing evidence, including a meta-analysis indicating that pre-IVF hysteroscopy particularly when performed immediately before treatment initiation can significantly enhance reproductive outcomes in asymptomatic patients (22). And another meta-analysis demonstrated that patients undergoing hysteroscopy prior to initiating IVF cycles had significantly higher clinical pregnancy rates compared to controls (OR 1.62, 95% CI 1.15–2.29; *I*^2^ = 62%). These findings suggest that hysteroscopy before IVF may enhance clinical pregnancy outcomes in first-time IVF attempts (23). Furthermore, Kamath demonstrated that pre-IVF screening hysteroscopy may improve clinical pregnancy rates, particularly benefiting women with two or more prior IVF failures based on subgroup analyses (24). Additionally, a previous systematic review has demonstrated that diagnostic hysteroscopy prior to IVF/ICSI may improve live birth rates (LBR) in women with recurrent implantation failure (RIF) compared to non-hysteroscopy controls. Through hysteroscopy, uterine lesions can be visually detected and corresponding treatments can be performed to improve endometrial receptivity, thereby increasing the clinical pregnancy rate. However, this review also suggests hysteroscopy provides no significant benefit when performed before a first IVF cycle (25). Moreover, a retrospective study suggested that hysteroscopy before transplantation did not improve the pregnancy rate (8). Nevertheless, these study's conclusions should be interpreted with caution as it did not account for potential confounding factors, particularly embryo quality—a critical determinant of reproductive outcomes. The study's analysis was limited to the number of embryos transferred. It did not extend to a comparison of embryo quality or a further analysis of the hysteroscopy subgroup. It is well-established in reproductive medicine that embryo quality represents the most critical determinant of successful implantation (26–29). So in consideration of embryonic factors, our multivariable regression analysis demonstrated that pre-IVF hysteroscopy and high-quality embryos were independent factors associated with a significantly higher clinical pregnancy rate. Furthermore, the small sample size and the retrospective nature of the study's may lead to statistical bias. Therefore, a high-quality randomized trial is necessary.

Our study revealed that approximately 50% of patients in the hysteroscopy group presented with abnormal uterine findings, predominantly endometrial polyps and chronic endometritis (CE). Existing research indicates that CE may contribute to recurrent implantation failure, while endometrial polyps can directly impair embryo implantation (18, 19). Currently, CE has emerged as a potential detrimental factor for fertility. Notably, reports indicate that between 14% and 67.5% of patients with recurrent implantation failure (RIF) are afflicted by CE (30, 31). In contrast, the non-hysteroscopy group lacked such diagnostic evaluation, leaving potential minor uterine pathologies undetected—factors that could equally compromise implantation success. The lack of a corresponding increase in the pregnancy rate among the non-hysteroscopy group in our study, despite a higher proportion of high-quality embryos, may be attributed to untreated subtle intrauterine pathologies. These pathologies could potentially lead to decreased endometrial receptivity. In addition, a randomized controlled trial study clearly demonstrated a significantly higher cumulative live birth rate in the hysteroscopy group than the control group during IVF. This study considers that uterine flushing can improve the embryo implantation rate by removing debris from tubes and changing the production of cytokines (32). Nevertheless, the limitation of this article is that it only takes into account the number of embryos, without analyzing whether there are differences in good-quality embryos. However, the quality of embryos is also a crucial factor in pregnancy. Our study systematically considers embryo quality assessment as an important parameter, thereby enhancing methodological rigor. Our multivariable regression analysis demonstrated a positive correlation of both embryo quality and hysteroscopy with the clinical pregnancy rate. Additionally, a recent RCT study demonstrated that hysteroscopic endometrial fundal incision during hysteroscopy before ET improved pregnancy rates (33). And previous investigations have demonstrated that diagnostic hysteroscopy can induce localized endometrial injury, which through synergistic multi-mechanistic pathways promotes decidualization and consequently enhances endometrial receptivity (34–37). These mechanisms may explain the lower pregnancy rates in the non-hysteroscopy group.

In the subgroup analysis of our research, it was shown that the clinical pregnancy rate was higher in the endometrial polyp group and CE group (compared with the non-hysteroscopy group). However, there was no statistically significant difference in the live birth rate. A potential explanation for these findings is the influence of embryo quality. Although hysteroscopic polypectomy and antibiotic treatment for CE can improve endometrial receptivity, these benefits may be offset by poor embryo quality, which can predispose to a higher risk of miscarriage. This interpretation is supported by the observed discrepancy in our data: the two subgroups exhibited lower rates of good-quality embryos and a concomitantly higher miscarriage rate compared to the control group.

While the clinical pregnancy rate in the intrauterine adhesion (IUA) group was comparable to that of the controls, it was associated with a lower live birth rate and a higher miscarriage rate. This discrepancy may be attributed to the endometrial fibrosis and scar tissue formation characteristic of IUA—a prevalent condition that causes infertility and recurrent pregnancy loss (38–40)—combined with the poorer embryo quality observed in this subgroup. Furthermore, the limited sample size may have introduced potential bias, as exemplified by the significantly lower AMH levels in the IUA group compared to controls, despite the absence of a difference in age.

From a cost-benefit perspective, it supports the hysteroscopy prior to IVF. The modest expense of hysteroscopy (≈1,000 RMB) must be weighed against the substantial cost of a failed IVF cycle (≈30,000 RMB) resulting from undiagnosed uterine factors. And in our study, hysteroscopy revealed that approximately half of the patients still had intrauterine abnormalities, which might lead to IVF failure. These patients with previously failed IVF cycles would then need to bear the additional financial burden of another IVF cycle (≈30,000 RMB) or frozen-thawed embryo transfer (≈4,000 RMB), along with the increased psychological stress.

The retrospective design of the study is the limitation of our study.

In conclusion, the results of our study indicated that it's necessary to perform diagnostic hysteroscopy before embryo transfer. Hysteroscopy enables the detection of minor uterine cavity lesions and the administration of corresponding treatments, thereby may enhance endometrial receptivity. However, randomized-controlled prospective trials are necessary to further understand the feasibility of performing hysteroscopy before embryo transfer.

## Data Availability

The original contributions presented in the study are included in the article/supplementary material, further inquiries can be directed to the corresponding author.
